# The AWED trial (Applying *Wolbachia* to Eliminate Dengue) to assess the efficacy of *Wolbachia*-infected mosquito deployments to reduce dengue incidence in Yogyakarta, Indonesia: study protocol for a cluster randomised controlled trial

**DOI:** 10.1186/s13063-018-2670-z

**Published:** 2018-05-31

**Authors:** Katherine L. Anders, Citra Indriani, Riris Andono Ahmad, Warsito Tantowijoyo, Eggi Arguni, Bekti Andari, Nicholas P. Jewell, Edwige Rances, Scott L. O’Neill, Cameron P. Simmons, Adi Utarini

**Affiliations:** 1 World Mosquito Program, Institute of Vector Borne Disease, Monash University, Melbourne, Australia; 2 Department of Biostatistics, Epidemiology and Population Health, Faculty of Medicine, Universitas Gadjah Mada, Yogyakarta, Indonesia; 3 Eliminate Dengue Project, Centre for Tropical Medicine, Faculty of Medicine, Universitas Gadjah Mada, Yogyakarta, Indonesia; 4 Department of Pediatrics, Faculty of Medicine, Universitas Gadjah Mada, Yogyakarta, Indonesia; 5 School of Public Health, University of California, Berkeley, USA; 6 Department of Health Policy and Management, Faculty of Medicine, Universitas Gadjah Mada, Yogyakarta, Indonesia

**Keywords:** *Wolbachia*, dengue, chikungunya, Zika, vector-borne disease, cluster randomised trial, test-negative design, Indonesia

## Abstract

**Background:**

Dengue and other arboviruses transmitted by *Aedes aegypti* mosquitoes, including Zika and chikungunya, present an increasing public health challenge in tropical regions. Current vector control strategies have failed to curb disease transmission, but continue to be employed despite the absence of robust evidence for their effectiveness or optimal implementation. The World Mosquito Program has developed a novel approach to arbovirus control using *Ae. aegypti* stably transfected with *Wolbachia* bacterium, with a significantly reduced ability to transmit dengue, Zika and chikungunya in laboratory experiments. Modelling predicts this will translate to local elimination of dengue in most epidemiological settings. This study protocol describes the first trial to measure the efficacy of *Wolbachia* in reducing dengue virus transmission in the field.

**Methods/design:**

The study is a parallel, two-arm, non-blinded cluster randomised controlled trial conducted in a single site in Yogyakarta, Indonesia. The aim is to determine whether large-scale deployment of *Wolbachia*-infected *Ae. aegypti* mosquitoes leads to a measurable reduction in dengue incidence in treated versus untreated areas. The primary endpoint is symptomatic, virologically confirmed dengue virus infection of any severity. The 26 km^2^ study area was subdivided into 24 contiguous clusters, allocated randomly 1:1 to receive *Wolbachia* deployments or no intervention. We use a novel epidemiological study design, the cluster-randomised test-negative design trial, in which dengue cases and arbovirus-negative controls are sampled concurrently from among febrile patients presenting to a network of primary care clinics, with case or control status classified retrospectively based on the results of laboratory diagnostic testing. Efficacy is estimated from the odds ratio of *Wolbachia* exposure distribution (probability of living in a *Wolbachia*-treated area) among virologically confirmed dengue cases compared to test-negative controls. A secondary per-protocol analysis allows for individual *Wolbachia* exposure levels to be assessed to account for movements outside the cluster and the heterogeneity in local *Wolbachia* prevalence among treated clusters.

**Discussion:**

The findings from this study will provide the first experimental evidence for the efficacy of *Wolbachia* in reducing dengue incidence. Together with observational evidence that is accumulating from pragmatic deployments of *Wolbachia* in other field sites, this will provide valuable data to estimate the effectiveness of this novel approach to arbovirus control, inform future cost-effectiveness estimates, and guide plans for large-scale deployments in other endemic settings.

**Trial registration:**

ClinicalTrials.gov, identifier: NCT03055585. Registered on 14 February 2017.

**Electronic supplementary material:**

The online version of this article (10.1186/s13063-018-2670-z) contains supplementary material, which is available to authorized users.

## Background

The health and economic impacts of arboviral diseases transmitted primarily by *Aedes aegypti* mosquitoes are escalating globally. In 2013, the estimated annual global burden of dengue was approximately 50–100 million clinically apparent dengue cases [1, 2] and approximately 10,000 deaths [2]. The burden of dengue has a cost of approximately $2.1 billion per year in the Americas [3] and almost $1 billion per year in Southeast Asia [4, 5]. Another epidemic arbovirus, the chikungunya virus (CHIKV), came to global attention in 2004 when it caused epidemics on several Indian Ocean islands before spreading to southern Europe and South and South East Asia. Like dengue, chikungunya is a febrile systemic viral illness of 4–7 days duration. Debilitating polyarthralgia can be a long-lasting sequelae of CHIKV infection [6]. In 2013, CHIKV emerged again in the Caribbean and caused epidemics in Latin American countries that are ongoing [7]. There are no licensed vaccines or specific therapies for chikungunya. Against this backdrop of endemic or epidemic dengue in over 100 countries, and recent explosive outbreaks of chikungunya, the Zika virus emerged in epidemic fashion in the Western Pacific in 2013 and in Latin America in 2015 [8]. As evidence accumulated that it causes congenital infections with severe outcomes, including fetal death and severe microcephaly, it was declared a public health emergency of international concern by the World Health Organization (WHO) [9]. Like chikungunya, there are no licensed vaccines or specific therapies for Zika.

Vector control targeted against *Ae. aegypti* is the mainstay of the fight against dengue, chikungunya and Zika disease transmission in endemic countries. Integrated control strategies include targeted residual spraying, space spraying, larval control and personal protection measures. However, successful broad-scale application of integrated vector control has been especially difficult to achieve in resource-limited endemic countries and impossible to sustain. Additionally, the evidence base to prioritise one intervention over another (e.g. larvicides and outdoor versus indoor insecticide space spraying) is weak as none have been robustly evaluated for impact on human infection and disease [10, 11]. Indeed, a recent meta-analysis of entomological intervention trials highlighted the paucity of reliable evidence for the effectiveness of any vector control method on dengue incidence [12]. Strikingly, none of the randomised controlled trials of vector control that were included in the meta-analysis investigated epidemiological impact (i.e. clinical disease endpoint) [12]. The difficulty of making evidence-based policy in relation to vector control has resulted in calls for improved trial methods [13].

The World Mosquito Program (formerly Eliminate Dengue Program) is an international research collaboration aiming to use *Wolbachia* to eliminate arboviral disease transmission by *Ae. aegypti* mosquitoes [14]. The presence of *Wolbachia* in *Ae. aegypti* mosquitoes renders them more resistant to disseminated arbovirus infection, including dengue, Zika, chikungunya and yellow fever viruses [15–17]. Thus, the critical and signature effect of *Wolbachia* as a public health intervention is to severely reduce the vectorial capacity of mosquito populations to transmit arboviral infections between humans. For field implementation, the approach works by seeding wild *Ae. aegypti* populations with *Wolbachia* via fortnightly releases, over a period of 2–3 months, of relatively small numbers of *Wolbachia*-infected mosquitoes. Over the subsequent 3–6 months, and through the actions of cytoplasmic incompatibility, the prevalence of *Wolbachia* in the local mosquito population increases, until such time as virtually all mosquitoes in the area carry *Wolbachia* [18]. Once established, *Wolbachia* sustains itself in the mosquito population for years. Of note, when *Wolbachia* is stably established in mosquito populations, there is predicted to be a 66–75% reduction in the basic reproduction number R_0_ for dengue viruses (DENV)-1 to -4 [19]. Reductions of this magnitude are predicted to result in local elimination of DENV transmission in most epidemiological circumstances [19].

With a population of approximately 250 million, Indonesia is the largest dengue endemic country in South East Asia. The administrative area of Yogyakarta City, in south-central Java, with a population of 414,000 (in 2016) in an area of 32 km^2^ [20], has generally had a higher dengue incidence than surrounding districts [21]. Between 2006 and 2016 the local public health surveillance system in Yogyakarta City received notification of 9418 hospitalised dengue cases, including large outbreaks in 2010 and 2016.

Small-scale proof-of-concept field trials of *Wolbachia* (*w*Mel strain) deployment have been successfully conducted in four small communities in districts adjacent to Yogyakarta City since 2014 (unpublished). The study protocol presented here represents the first cluster randomised trial to experimentally measure the efficacy of *Wolbachia* in reducing DENV transmission.

## Methods/Design

The aim of this study is to determine whether large-scale deployment of *Wolbachia*-infected *Ae. aegypti* mosquitoes leads to a measurable reduction in dengue incidence in people living in treated (intervention) areas, compared to those in untreated areas. The primary endpoint is symptomatic, virologically confirmed DENV infection of any severity. Secondary objectives are the measurement of the efficacy of the *Wolbachia* method in reducing the incidence of symptomatic, virologically confirmed Zika virus (ZIKV) or CHIKV infection of any severity in treated areas, relative to untreated areas; quantification of the level of human mobility within Yogyakarta City and estimation of the proportion of time that residents spend outside the treatment arm to which they were randomised; and measurement of the impact of *Wolbachia* deployment on the prevalence of arbovirus-infected *Ae. aegypti* mosquitoes.

### Study design

The study is a parallel, two-arm, non-blinded cluster randomised controlled trial conducted in a single site in Yogyakarta, Indonesia. The study area was subdivided into 24 contiguous clusters of roughly equal area and population size, which were randomly allocated in a 1:1 ratio to receive *Wolbachia* deployments or no intervention. Additional file 1 provides an overview of the elements of the study protocol, as described in a SPIRIT checklist.

The impact of *Wolbachia* deployments on dengue incidence will be assessed using a novel epidemiological study design, the cluster-randomised test-negative design (CR-TND) trial, which is described in detail elsewhere [22, 23]. In brief, dengue cases and arbovirus-negative controls will be sampled concurrently from within the population of patients presenting with undifferentiated febrile illness to a network of primary care clinics across the study area, with case or control status classified retrospectively based on the results of laboratory diagnostic testing. Efficacy is estimated by comparing the exposure distribution (probability of living in a *Wolbachia*-treated area) among virologically confirmed dengue cases against the exposure distribution in test-negative controls. The distribution of *Wolbachia* exposure in the sampled arbovirus-negative controls is assumed to reflect the distribution of *Wolbachia* exposure in the underlying source population that gave rise to cases, as long as a core assumption is met that the relative propensity to seek healthcare for undifferentiated febrile illness at a primary health clinic (known locally as *Puskesmas*), in intervention versus untreated arms, is the same for dengue cases as for other febrile illness controls [23]. This should be upheld if dengue cases and other undifferentiated febrile illness controls are clinically indistinguishable until laboratory diagnosis. The concurrent sampling of controls and cases means that the odds of *Wolbachia*-exposure among sampled dengue cases relative to test-negative controls (odds ratio), is an unbiased estimate of the relative incidence of medically attended dengue in *Wolbachia-*treated versus untreated clusters (risk or rate ratio (RR)), from which protective efficacy can be directly estimated [23]. The null hypothesis is that the relative incidence of virologically confirmed dengue in *Wolbachia*-treated and untreated areas is one. If *Wolbachia* has a protective effect against DENV transmission, we would expect the RR for virologically confirmed dengue in *Wolbachia*-treated areas compared to untreated areas to be below one.

### Study setting

**Fig. 1:**
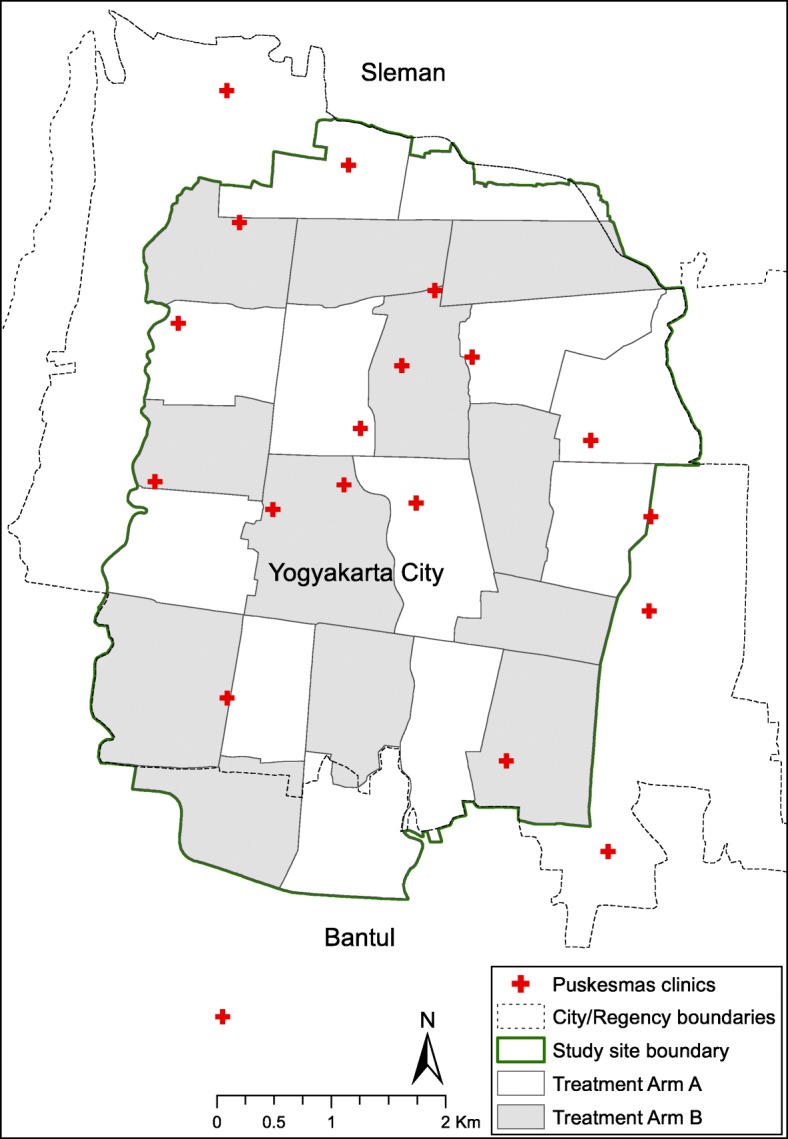
Map of Applying *Wolbachia* to Eliminate Dengue (AWED) trial study site. The study site is a continuous area of 26 km^2^, including 24 km^2^ in Yogyakarta City and 2 km^2^ in adjacent Bantul Regency, to the south. Nineteen government primary healthcare clinics (*Puskesmas*) where recruitment of febrile participants will take place are shown

The study will be conducted in the adjacent districts of Yogyakarta City and Bantul, in the province of Yogyakarta Special Region, Indonesia. Yogyakarta City has an area of 32 km^2^ and had a population of approximately 414,000 in 2016 [20]. The study site area is of 26 km^2^, including 24 km^2^ within Yogyakarta City and extending into 2 km^2^ of the adjacent administrative area, Bantul District, to the south of Yogyakarta City (Fig. 1). The study site is a continuous urban area, with a total population of approximately 350,000 and an average population density of 13,460 persons per km^2^. The annual notified dengue incidence rate in Yogyakarta ranged between 91 and 412 cases per 100,000 population during the years 2006–2016.

The study site was subdivided into 24 contiguous clusters, each with an area of approximately 1 km^2^ (range 0.7–1.65 km^2^). There are no buffer areas between clusters, but natural borders (roads, rivers, non-residential areas) were used to define cluster boundaries as much as possible, to limit the spatial spread of *Wolbachia* from treated clusters into untreated areas, and of wild-type mosquitoes in *Wolbachia*-treated clusters. Exclusion areas were minimised, and any areas within the study site where releases are not possible for reasons of logistics, public acceptance or absence of mosquito populations (e.g. hospitals, public space, open parkland) were pre-specified prior to randomisation and balanced between study arms. No attempt will be made to alter the routine dengue prevention and vector control activities conducted by public and private agencies throughout the study area (treated and untreated clusters).

### Randomised allocation of the intervention

Among the 24 clusters, 12 were randomised to receive *Wolbachia* deployments and 12 to remain untreated (Fig. 1). Covariate constrained (‘restricted’) randomisation was used to prevent a chance imbalance in the baseline characteristics or spatial distribution of treated and untreated clusters, given the relatively small number of randomisation units, using the method outlined by Hayes and Moulton [24]. Constraining variables include those that may be potentially confounding covariates, may impact sample size or are relevant for logistics (Table 1).

**Table 1:** Covariates included in constrained randomisation

Category	Covariate	Rationale	Balancing criterion
Potential confounders	1. Age: % of population < 15 years^a^	Dengue risk is age dependent	Each arm within ± 5% of overall population value
	2. 3-year average dengue incidence rate^a^	Historical dengue incidence may predict future risk	Each arm within ± 5% of overall population value
	3. Education: % completed high school^a^	Proxy for socioeconomic status that may predict dengue risk	Each arm within ± 5% of overall population value
Potential sources of bias	4. Incidence of other febrile illness^f^ presenting to *Puskesmas* clinics in 2014–2015^b^	Prevent chance association between other febrile illness and intervention	Each arm within ± 5% of overall population value
Sample size	5. Number of clusters	To maximise precision and power	12 clusters per study arm
	6. Cluster population^c^	To maximise precision and power	Each arm 45–55% of total population
Logistics	7. Total cluster area (km^2^)^d^	Releases to be done over approximately half the city	Each arm 45–55% of total area
	8. Non-release area within cluster (km^2^)^e^	To prevent an excess of non-residential areas falling in intervention arm	Each arm 45–55% of total non-release area
	9. Four spatial strata	To prevent a large contiguous intervention area	Within each spatial stratum, three clusters per study arm

Data sources: ^a^Yogyakarta and Bantul District Health Offices; ^b^Records from individual primary health clinics (Puskesmas); ^c^Statistics Indonesia (BPS), 2015; ^d^Calculated in ArcGIS; ^e^Calculated in ArcGIS and Google Earth.

^f^Other febrile illness extracted based on ICD10 codes R50 (Fever of other and unknown origin), R50.9 (Fever, unspecified), A75.9 (Typhus fever, unspecified), A49 (staphylococcal infection, unspecified site)

The covariates, data sources and cluster values included in the constrained randomisation are summarised in Table 1. Briefly, values for each balancing covariate were calculated for each of the 24 clusters and across the study area as a whole. Stata statistical software (v13.0, StataCorp, USA) was used to generate a large number (*n* = 100,000) of potential random allocations of the 24 clusters equally into two study arms. For each allocation, the value of each balancing criterion was calculated in each study arm in two ways, namely (1) the average of the 12 cluster-level values and (2) the aggregate arm-level value. Each potential random allocation was evaluated against the pre-defined balancing criteria (Table 1), and rejected if they were not met. All potential allocations that satisfied the balancing criteria were retained (*n* = 247), and a single allocation was then randomly selected from within the restricted list of balanced allocations. Finally, a coin toss was used to determine which of the two study arms (A or B) was to receive *Wolbachia* releases. There were thus 494 possible distinct intervention allocations, exceeding the guideline threshold of 100–150 allocations suggested by others [24–26] and shown in extensive simulations to achieve excellent performance in terms of confidence interval coverage [22]. The set of potential constrained randomisations was examined to assess how frequently a pair of clusters appeared in the same arm, and the pairwise correlation in cluster-level dengue incidence over 10 years was assessed, in accordance with the validity checks recommended by Hayes and Moulton [24]. The two pairs that occurred most frequently in the same arm (> 65% of potential allocations) actually had less than typical correlation when compared to other pairs.

Randomisation was conducted in January 2017 after completing an extensive community engagement process and obtaining written agreement from the leaders of the 37 administrative areas (*kelurahans*) indicating that they were willing to have releases occur in their area, if randomly allocated. The final stages of the randomisation process, including the selection of one allocation from a list of 100 balanced possibilities (randomly sub-selected from the 247 total balanced allocations) and the coin toss to determine which arm received the intervention, were conducted in a public forum to which community leaders and other key stakeholders were invited to maximise transparency and acceptance of the process.

### *Wolbachia* deployment strategy

*Wolbachia*-infected *Ae. aegypti* will be deployed by setting mosquito release containers (MRCs) in residential and non-residential properties throughout the intervention clusters. An MRC is a small plastic tub containing *Ae. aegypti* eggs, Tetramin food and water, placed in an outdoor area. Adult mosquitoes emerge from small holes in the side of the MRC, approximately 7–12 days after the MRC is deployed. Based on previous field work, we expect to release 30,000–150,000 adults per km^2^ during each release week.

*Wolbachia* will be deployed through rolling releases across intervention clusters within a 6–9 month period, with the aim of achieving a high prevalence of *Wolbachia* in treated clusters within a maximum of 12 months (from the start of the release). Once the cluster-level *Wolbachia* prevalence in trapped *Ae. aegypti* reaches 60–80%, deployment will then stop in that cluster and monitoring of *Wolbachia* prevalence in trapped mosquitoes will continue throughout the study period. There will be no remediation with additional releases if *Wolbachia* prevalence drops below 60% in the future.

### *Wolbachia* monitoring strategy

A network of BG-Sentinel adult mosquito traps (BioGents, Germany) will be established throughout intervention clusters prior to the commencement of releases, evenly spaced throughout residential areas at a density of approximately 16 traps per km^2^. A BG trap network of the same density (16 traps per km^2^) will be established also in untreated clusters prior to the commencement of the clinical study. BG-Sentinel traps will be serviced weekly, with trapped mosquitoes screened for *Wolbachia* at weekly, fortnightly or 4-weekly intervals throughout the duration of the trial, depending on the stage of release and establishment. Once *Wolbachia* has established in treated clusters and the clinical study has commenced, *Wolbachia* screening will occur 4-weekly throughout the remainder of the study period.

Trapped mosquitoes will be identified using microscopy. Individual mosquitoes (male and female) will be tested for *Wolbachia* by quantitative polymerase chain reaction (PCR) assay. The *Wolbachia* prevalence in screened *Ae. aegypti* will be reported aggregated to the cluster level.

For the purposes of measuring the efficacy endpoint in the primary intention-to-treat analysis, *Wolbachia* will be considered as established throughout intervention clusters 1 month after completing releases in the last cluster.

### Study participants

Participant enrolment to measure the efficacy endpoint will be conducted at a network of 19 *Puskesmas* throughout the study site (Fig. 1). Based on 2 years of historic data collated from the *Puskesmas* network in the study area, it is estimated that at least 5000 patients per year present to these clinics with febrile illness (range 200–1500 per clinic per annum). We will invite the participation of all patients aged 3–45 years presenting to any of the participating clinics with undifferentiated fever of 1–4 days duration, who meet the eligibility criteria as described in Table 2 and who provide written informed consent (from a parent or guardian for participants aged < 18 years). An individual presenting on repeat occasions for different febrile episodes will be eligible for enrolment during each different episode. However, an individual may only be enrolled once during a single illness episode, which is defined conservatively as illness occurring within 4 weeks of a previous febrile illness.

**Table 2:** Inclusion and exclusion criteria for study enrolment

Inclusion Criteria *(Participants must meet all of the following)*	Exclusion Criteria *(Participants will not be eligible for enrolment if any of the following are identified)*
Fever, either self-reported or measured at enrolment	Prior enrolment in the study within the previous 4 weeks
Date of onset of fever between 1 and 4 days prior to the day of presentation	Localising features suggestive of a specific diagnosis other than an arboviral infection, e.g. severe diarrhoea, otitis, pneumonia
Aged between 3 and 45 years old	
Resided in the study area every night for the 10 days preceding illness onset	

### Data and sample collection

**Fig. 2:**
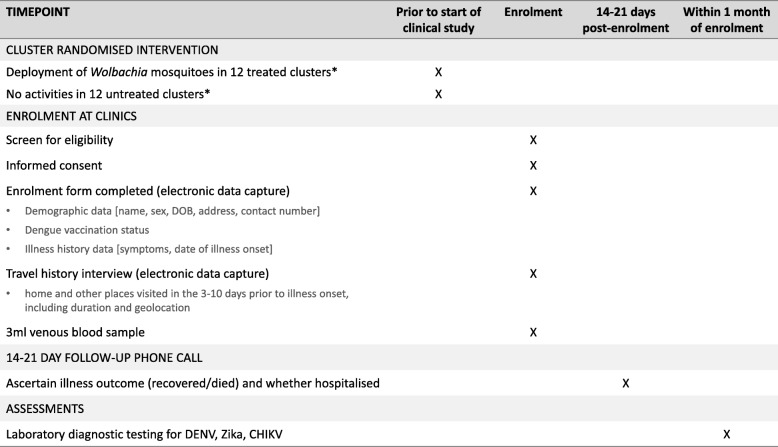
Schedule of enrolment, data collection and assessments (SPIRIT Figure) *Routine dengue prevention and vector control activities will not be altered in treated or untreated clusters

A unique identifier will be assigned to each participant at enrolment. Basic demographic details, eligibility against the inclusion criteria, illness onset date and a 10-day retrospective travel history will be recorded in a standardised electronic data collection form. Figure 2 summarises the data and samples to be collected from each participant. Data and samples are collected at a single time point at enrolment, with no longitudinal follow-up of participants except for a phone call to establish their status at 14–21 days post enrolment.

A brief travel history interview will be conducted at enrolment to determine the main places visited by each participant on days 3–10 prior to illness onset, i.e. during the incubation period for dengue. Participants will be prompted to recall the locations visited for an hour or more at a time between 5 am and 9 pm on each day, and the duration spent at each location, using a tablet-based data collection tool. The coordinates of each unique location visited will be derived by geolocation on a digital map. These data will be used to determine the proportion of time spent in *Wolbachia*-treated and untreated areas, for the per-protocol analysis.

A single 3 mL venous blood sample will be collected from all consenting participants on the day of enrolment. Blood samples will be collected and transferred to the project laboratory on the day of collection. All diagnostic specimens will be processed and stored on the same day as sample receipt and plasma stored at –80 °C until testing.

All enrolled participants will be contacted by telephone 14–21 days post enrolment to ascertain their health status, recorded categorically as recovered or died, and whether or not they were ever hospitalised during this illness. This is for the purpose of ascertaining the safety endpoints (see page 22), and does not apply to the primary or secondary outcomes of the study. For any participant uncontactable after three attempts, the status will be recorded as unknown. Any death of a study participant within 14–21 days of enrolment will be classified as a serious adverse event and reported to the trial steering committee (TSC), ethical committees and independent data monitoring committee (IDMC) within 7 days of ascertainment. The proportion of participants in each arm that were hospitalised or died will be reviewed at each meeting of the IDMC.

### Laboratory investigations

**Fig. 3:**
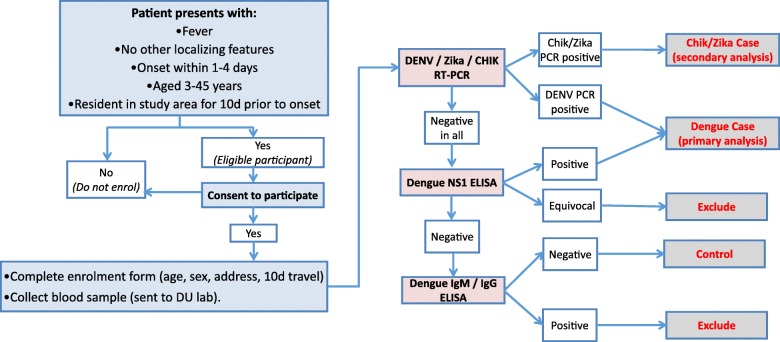
Flowchart of data and sample collection and diagnostic algorithm. Blue boxes indicate participant recruitment and enrolment activities undertaken at *Puskesmas* clinics, including screening against inclusion/exclusion criteria, obtaining written informed consent, and collection of demographic and travel history data and a blood sample. Pink boxes indicate the laboratory diagnostic testing to be performed at the project laboratory (DU), the results of which (white boxes) will be used to classify participants as virologically confirmed dengue, Zika or chikungunya cases, arbovirus-negative controls, or excluded due to inability to rule out arbovirus infection (grey boxes) according to the algorithm shown

Reverse transcriptase quantitative PCR (RT-qPCR) is the gold standard method of diagnosing arboviral infections in the first few days of illness. We will use an internally controlled triplex RT-qPCR assay to detect DENV, CHIKV and Zika viruses in plasma samples from all enrolled participants (Fig. 3). Dengue non-structural protein 1 (NS1) Platelia ELISA (BioRad, USA) and IgM and IgG capture ELISA (Panbio, Australia) will be performed according to the manufacturers’ instructions. These kits were selected as they were among the best performers in a WHO evaluation of dengue diagnostic tests [27, 28].

Diagnostic test results will not be reported back to individual participants since batch processing of samples will mean that results are not available in time to inform clinical management. Participants will be managed according to standard clinical practice by the treating clinicians.

### Case and control classification

The diagnostic algorithm for classifying dengue, Zika or chikungunya cases and arbovirus-negative controls is shown in Fig. 3. Dengue cases are defined as patients with virologically confirmed DENV infection, meeting the clinical criteria for enrolment and also with a positive result in NS1 ELISA and/or DENV RT-qPCR.

Controls are patients meeting the clinical criteria for enrolment, but with negative test results for CHIKV RT-qPCR, Zika RT-qPCR, DENV NS1 ELISA, DENV RT-qPCR, and DENV IgM and IgG ELISA.

For the secondary endpoints, Zika or chikungunya cases are defined as patients with virologically confirmed Zika virus or CHIKV infections, meeting the clinical criteria for enrolment and also with a positive result in Zika RT-qPCR or CHIKV RT-qPCR, respectively, and controls are defined as above.

### Expected study duration

**Fig. 4:**
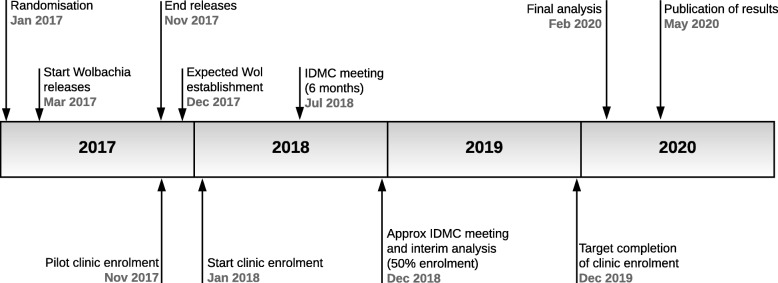
Applying *Wolbachia* to Eliminate Dengue (AWED) trial time line. *Wol Wolbachia*, *IDMC* Independent Data Monitoring Committee

*Wolbachia* deployments commenced in March 2017 and finished in November 2017. The clinic-based sampling of febrile patients commenced in a pilot phase in November 2017, with enrolment into the intention-to-treat dataset commencing in January 2018. The study timeline is depicted in Fig. 4. Participant enrolment will continue for 2 years, or longer if required to attain the minimum sample size for intention-to-treat analysis. Recruitment will continue for 24 months even if the estimated minimum sample size is reached sooner.

### Power calculations

It is estimated that approximately 1000 cases plus four times as many controls will be sufficient to detect a 50% reduction in dengue incidence with 80% power. The estimate relies on several assumptions, outlined below. Sample size requirements will be re-estimated using observed data after 50% of the target recruitment is completed to account for possible violations to these assumptions. This power reassessment will be based on the actual observed distributions of test-positive (i.e. dengue) and test-negative (i.e. other febrile illness) cases across the 24 clusters, and will then be used to make recommendations regarding the necessary sample size of total dengue case counts and whether extending eligible enrolments beyond the current protocol may be desirable.

No formulae have previously been published to estimate sample size for the proposed study design, i.e. a cluster randomised trial with a test-negative design, where the intervention effect is estimated from outcome-based sampling of test-positive and test-negative patients and ascertainment of their exposure status. Randomisation provides a basis of inference in comparing intervention clusters with control clusters as, under the null hypothesis, there should be no difference with regard to the relative appearance of test positives and negatives in clusters, on average, across the two arms. Thus, we have proposed, as the primary analytical approach, a comparison of the exposure odds among test-positive cases versus test-negative controls (for data aggregated across all clusters), with the null hypothesis that the odds of residence in a *Wolbachia*-treated cluster is the same among test-positive cases as test-negative controls [22, 23]. The resulting odds ratio thus provides an estimation of the intervention effect and, as demonstrated previously, provides an unbiased estimate of the relative risk provided that the key assumptions underlying the TND are upheld.

A secondary approach employs, as a summary measure for a group-level analysis, the proportion of test-positive cases amongst all tested participants in each cluster, with a comparison of the average of these proportions in the intervention arm versus the untreated arm forming the basis of hypothesis testing for intervention effect. The null hypothesis is that the average proportion of total enrolled participants that are cases is the same in treated and untreated study arms. The alternative hypothesis is that the proportion of enrolled participants that are cases is lower in the *Wolbachia*-treated arm than the untreated arm.

Simulations were used to estimate the power to detect a range of intervention effect sizes using the two methods above, assuming 12 clusters per arm, a total of 1000 true dengue cases enrolled and 4000 non-dengue controls, and using empirical data on population, historical dengue incidence and incidence of other febrile illness in the 24 study clusters (including the observed spatial distribution of dengue and other febrile illnesses among clusters) to define the baseline characteristics for the simulated scenarios [22]. We randomly allocated half the clusters to receive the intervention; this random allocation was repeated one million times, and only those allocations were kept in which the balancing criteria specified in the constrained randomisation methods were met (*n* = 247 balanced allocations, and thus 494 possible distinct randomisations of intervention allocation). Dengue case numbers per cluster were either kept at baseline values (for the simulation at the null; i.e. RR = 1) or reduced proportionately (for simulations of intervention effects of RR = 0.6, 0.5, 0.4, 0.3). For each of these five ‘true’ effect sizes, applied to each of the 247 balanced allocations, the ‘observed’ effect size was calculated from the simulated data by the two methods outlined above, namely (1) aggregated odds ratio for residence in a treated cluster among cases versus controls and (2) difference of the average cluster summary proportions (cases/cases+controls) between study arms (compared using a standard *t* test). Statistical inference, from the *t* test directly, or, for the odds ratios, using permutation distribution approximations with standard errors adjusted to account appropriately for the clustered nature of the data [22], respectively, was used to calculate the proportion of constrained random allocations that yielded a significant result. This provided an estimate of Type I error at the null, and power away from the null (Table 3). Both of these approaches thus are using approximations to the exact permutation distribution [22]. In practice, the appropriate reference distribution for inference will be based on the set of 247 potential balanced allocations.

**Table 3:** Percentage of random allocations that yield significant results on simulated data (i.e. power)

Risk ratio	*t* test		Odds ratio test	
	Constrained	Random	Constrained	Random
1	0.13	5	1	7
0.6	48	49	61	57
0.5	81	75	89	82
0.4	97	93	99	96
0.3	100	99	100	100

The results show that constrained randomisation is somewhat conservative at the null but generally increases power moderately. The odds ratio test is more powerful than the *t* test approach, and will thus be used as the primary analysis with the additional attraction of being standard for the traditional test-negative design.

### Statistical analyses

#### Primary endpoint – the impact of *Wolbachia* deployment on dengue

The intention-to-treat (primary) analysis will consider *Wolbachia* exposure as a binary classification based on residence in a cluster allocated to *Wolbachia* deployment or not. Residence will be defined as the primary place of residence during the 10 days prior to illness onset. The intervention effect will be estimated from an aggregate odds ratio comparing the exposure odds (residence in a *Wolbachia*-treated cluster) among test-positive cases versus test-negative controls (for data aggregated across all clusters), using the constrained permutation distribution as the foundation for inference. The null hypothesis is that the odds of residence in a *Wolbachia*-treated cluster are the same among test-positive cases as among test-negative controls. The resulting odds ratio provides an unbiased estimate of the RR provided that the key assumptions underlying the TND are upheld. Of note, since the constrained permutation distribution used for statistical inference contains only the 247 potential allocations (494 distinct randomisations) that meet all balancing criteria, the most extreme odds ratio in the distribution would carry a two-sided *p* value of approximately 0.004 (2/494). Therefore *p* < 0.004 is the minimum threshold at which statistical significance can be evaluated in this design.

A secondary group-level analysis will be performed using a cluster-level summary measure of the proportion of test-positive individuals amongst all tested individuals in each cluster. The difference in the average proportion of test positives between the intervention clusters and untreated clusters will be used to test the null hypothesis of no intervention effect using the *t* test statistic but basing inference on the exact permutation distribution. These average proportions in each arm can be used to derive an estimate of the RR of dengue in treated versus untreated clusters, which is a more intuitive effect measure [22]. Briefly, we can substitute the estimated difference in the proportions, *d*, into the formula:

$$ d=\frac{1}{1+\left(\frac{r}{2}\right)\left(1+ RR\right)}-\frac{RR}{RR+\left(\frac{r}{2}\right)\left(1+ RR\right)} $$

where *r* is the overall ratio of test negatives to test positives, which yields a quadratic equation for the unknown *RR.* Only one solution is plausible so that this then yields an estimate of *RR*, along with the appropriately transformed confidence interval (from that associated with *d*).

The per-protocol analysis will consider *Wolbachia* exposure as a quantitative index based on measured *Wolbachia* prevalence in local *Ae. aegypti* mosquitoes in the cluster of residence and other locations visited by the participant during a period of 3–10 days prior to illness onset, as reported in the travel history interview. A weighted ‘*Wolbachia* exposure index’ (WEI) will be defined for each participant, as follows. The aggregate *Wolbachia* prevalence for each cluster will be calculated each month from all *Ae. aegypti* trapped in that cluster. The WEI for each participant will then be calculated by multiplying the cluster-level *Wolbachia* prevalence (in the month of participant enrolment) at each of the locations visited, by the proportion of time spent at each location, to give a value on a continuous scale from 0 to 1. Cases and controls will be classified by strata of their WEI (e.g. 0–0.2, 0.2–0.4, 0.4–0.6, 0.6–0.8, 0.8–1). This acknowledges that the WEI is not a highly precise measure, and serves to reduce error in exposure classification. The per-protocol analysis therefore allows for *Wolbachia* exposure to vary in a location over time, and also accounts for human mobility. This analysis can also account for the temporal matching of dengue cases and test-negative controls, where risk sets of cases and controls will be defined by frequency matching enrolled confirmed dengue cases to arbovirus-negative controls with illness onset in the same calendar month. In the unlikely event that a minimum of four controls cannot be found for a case within the same calendar month, the window for matching can be extended until four controls are identified, for that case only. Both inference methods described above for the intention-to-treat analysis will be extended to allow for this individual level covariate using regression approaches and extension of the permutation-derived inference used to test the null [29]. For a time-adjusted analysis, a Cox proportional hazard model will be fitted, incorporating the temporal risk sets using a shared frailty for cluster membership. Such models yield an estimate, and associated confidence interval, for the incidence rate ratio (the relative hazard). The WEI strata will be included as categorical variables to calculate stratum-specific incidence rate ratios (relative to the baseline 0–0.2 stratum). This will allow examination of a ‘dose response’ relationship. An additional benefit of transforming WEI to a categorical variable is that it avoids any assumption of linearity in the dose response relationship.

The per-protocol analysis will include all participants enrolled from the commencement of the main phase of clinic-based sampling (i.e. excluding the pilot phase, but including participants enrolled before *Wolbachia* was established in treated clusters).

#### Secondary endpoint – the impact of *Wolbachia* deployment on Zika and chikungunya

There exists no baseline data on the prevalence of Zika or chikungunya infection among febrile patients presenting to primary healthcare clinics in Yogyakarta City from which to estimate the expected number of cases; therefore, these secondary analyses are exploratory only and not subject to any formal sample size or power calculations. Blood samples from enrolled participants will be tested by Zika and chikungunya PCR for the purpose of defining arbovirus-negative controls for the primary analysis, as described above. These results will permit estimation of the prevalence of virologically confirmed Zika virus and CHIKV infection among the study population of ambulatory febrile patients presenting to primary healthcare.

If virologically confirmed Zika or CHIKV cases are detected, a secondary analysis will estimate the efficacy of *Wolbachia* deployments in reducing the incidence of symptomatic virologically confirmed Zika virus and CHIKV infection. The same enrolled patient population will be used to analyse all three arbovirus endpoints (dengue, Zika and chikungunya), and the same statistical methods for intention-to-treat and per-protocol analyses will be used as described for the primary (dengue) endpoint above. For Zika and chikungunya, the cases will be defined as enrolled participants who test positive by Zika or chikungunya PCR, respectively, and the controls will be those who test negative to all three arboviruses.

#### Secondary endpoint – the impact of *Wolbachia* deployment on routine dengue case notifications

The existing system for routine notification of dengue cases in Yogyakarta City is based on hospital reporting of cases diagnosed clinically as dengue haemorrhagic fever, which historically have not been accompanied by supportive laboratory testing. Since March 2016, hospitals have been encouraged to record a serological testing result, where available, on the report form, and also to report cases diagnosed clinically as dengue fever where there is a confirmatory NS1-positive test result. A separate reporting system, established in March 2016, collates data on the number of NS1 rapid tests performed and the number of positive tests, in *Puskesmas* across the city.

We will collate data from these two reporting systems on a monthly basis from 2016 to 2020, aggregated by *Kelurahan* of residence, to monitor trends in reported dengue incidence across the City and by *Kelurahan*, before, during and after *Wolbachia* deployment.

#### Secondary endpoint – human mobility in Yogyakarta and implications for measuring efficacy of *Wolbachia* deployment

Understanding the level and distribution of routine movements among the study population is critical to the success of this study design. ‘Contamination’ by human mobility between study arms may lead to a dilution of the true intervention effect in the intention-to-treat analysis, and will influence the degree to which the per-protocol analysis can retain comparison groups with different levels of *Wolbachia* exposure after taking into account participants’ crude movement patterns. The data captured through the travel history interview will be analysed to quantify the geographical extent and duration of participants’ travel outside the home, and to estimate the proportion of daytime (‘at risk’) hours that participants randomised to treated and untreated arms actually spend in *Wolbachia-*treated and untreated areas, overall and by age group. An age-stratified analysis will describe the proportion of participants’ time (5 am to 9 pm) spent at home versus away from home, estimate the distribution of participants’ time as a function of increasing distance from home, and identify the predominant non-home locations at which participants in different age groups spend their daytime hours. This information can inform the design of future trials of cluster-randomised household-based interventions by estimating the optimal size of the clusters needed to account for the majority of daily movements and providing information on the degree to which a true intervention effect might be diluted by movement of participants between treatment arms.

#### Secondary endpoint – the impact of *Wolbachia* deployment on the prevalence of arbovirus-infected *Ae. aegypti* mosquitoes

We will test the hypothesis that *Wolbachia* deployments will reduce the prevalence of arbovirus-infected *Ae. aegypti* mosquitoes. In this context, we interpret arbovirus-infected *Ae. aegypti* mosquitoes as a proxy for the presence of a viremic human host in close proximity to the location of the infected mosquito. To test this hypothesis, all *Ae. aegypti* mosquitoes caught in the network of BG-Sentinel traps in the study area will be tested by PCR for the presence of DENV, CHIKV and Zika virus. Assuming a DENV infection prevalence of 0.1% in untreated clusters during the 2-year study period 2018–2019 (based on previous testing of 29,000 wild-type female *Ae. aegypti* in Nov 2015 to May 2016), and assuming independence, then testing a total of 30,500 mosquitoes per arm would give 80% power to detect a 60% reduction in DENV prevalence in treated clusters (one-sided z-test; G*Power version 3.1.9.2 [30]).

### Monitoring of unintended adverse effects of *Wolbachia* releases

In order to demonstrate that the deployment is not associated with any excess of a severe adverse outcome, we will follow-up all enrolled participants (test-positive cases and test-negative controls) by telephone 14–21 days post enrolment to ascertain their health status, recorded categorically as recovered/died, and whether or not they were ever hospitalised during this illness. Any death of a study participant within 14–21 days of enrolment will be classified as a serious adverse event and reported to the TSC, IDMC and the ethics committees of Universitas Gadjah Mada and Monash University within 7 days of ascertainment. The proportion of participants that were hospitalised or died in each arm will be reviewed by the IDMC each time they meet, and at any other time at the request of the TSC or other agencies.

### Data management

Data collected from participants in the clinical study will be entered directly in standardised electronic data capture forms and digital mapping interfaces, deployed through web-based applications on mobile devices, and stored directly in a custom designed web-hosted relational database. Laboratory diagnostic results will be captured directly from laboratory assay output, and uploaded to the same database.

In order to maintain blinding of research staff and data managers, measures will be put in place to ensure the datasets identifying participants’ exposure status (cluster of residence and clusters visited during 10 days prior to illness) will remain unlinked and partitioned from the dataset that classifies their case/control status until the final analysis. In the event that the IDMC requires data to be unblinded following the interim analysis, a single member of the World Mosquito Program, Monash University, data management unit will be responsible for linking the participant dataset to the exposure status.

Role based, tiered access permissions will be used to control access to the trial database and associated data capture applications. User logs will document the activities of all users. Validity controls will be applied at the point of data capture into electronic forms by predefining value ranges, specifying categorical option lists and minimising the use of free text fields. Quality control in the form of logic and consistency checks will be applied at several stages of data capture and management, including (1) at the point of data capture into an electronic form, (2) at the point of upload into the web-based database and (3) during routine monitoring processes by internal and external data monitors. An audit trail will be preserved within the database to capture the history of any changes made to data records after their initial capture. Data acquisition, data management and independent monitoring procedures, as well as the indefinite retention (and for a minimum of 5 years after study completion) of data relating to the trial, including field entomology and epidemiological data, are in accordance with International Council for Harmonization on Good Clinical Practice guidelines and requirements.

### Trial governance

The Principal Investigator from Universitas Gadjah Mada, Yogyakarta, supported by the Chief Investigator from Monash University, will be responsible for ensuring the study is performed in compliance with the approved protocol and the principles of Good Clinical Practice.

The TSC will provide overall supervision of the trial, including monitoring of recruitment progress, and will consider and act upon (as appropriate) any recommendation from the IDMC with regards to early stopping of the trial.

The Trial Operations Group will, under the delegation of the Principal Investigator, be responsible for day-to-day coordination of the trial processes.

The Monitoring Group will be independent of the investigators, and will conduct periodic monitoring of adherence to regulatory requirements, and implementation of study processes including data collection and storage, sample collection and chain of custody, and laboratory processes.

The Data Analysis Working Group will be chaired by the trial statistician, and will be responsible for developing the statistical methods for randomisation, data cleaning and validation, and preparing and implementing the statistical analyses.

An IDMC will be constituted from local and international experts in accordance with standard practice for randomised clinical trials. The IDMC will meet at study initiation, 6 months following the commencement of clinic-based enrolment, and at 50% enrolment of the estimated minimum required number of dengue cases (*n* = 500), as well as any other time at the request of the TSC or other agencies. Their primary role is to safeguard the interests of the trial participants, to assess the safety and efficacy of the intervention during the trial, and to monitor the overall conduct of the trial.

The IDMC will provide recommendations about stopping or continuing the trial, and may also make recommendations relating to trial procedures, and data management and quality control. Any proposed major modifications to the study protocol will be reviewed by the IDMC, and approval for a protocol amendment will be sought from the relevant institutional review boards (IRBs) prior to their implementation. Detailed responsibilities and terms of reference will be set out in an IDMC charter and agreed to by all IDMC members prior to study commencement.

### Interim analyses and stopping rules

An interim analysis of the primary endpoint (intention-to-treat analysis only, using the odds ratio approach based on the permutation distribution and blinded to treated and untreated study arms) will be conducted by the trial statistician when enrolment reaches 50% of the estimated minimum required number of dengue cases (*n* = 500).

The IDMC may recommend modification or termination of the study if analyses of data from the first 50% of the estimated minimum required number of dengue cases indicate beyond reasonable doubt that exposure to *Wolbachia* confers a reduced risk of dengue in the intention-to-treat analysis. As detailed in the analysis methods, the use of the constrained permutation distribution for statistical inference means the smallest two-sided *p* value that can be observed is approximately 0.004. The usual Haybittle–Peto boundary [31], requiring *p* < 0.001 at interim analysis to consider stopping for efficacy, cannot therefore be applied precisely. Instead, *p* < 0.01 at interim analysis will be used as guidance for considering stopping early for efficacy. The IDMC may also recommend termination if preliminary data clearly suggest that *Wolbachia* is associated with an excess of dengue (or Zika or chikungunya) cases. A less conservative *p* < 0.05 in the direction of harm will be used as a guidance. Termination or modification may also be recommended for any other operational reason (e.g. participant enrolment rates), perceived safety concern or external factor. The final decision to terminate or modify the study rests with the TSC.

### Ethical considerations

The study protocol (version 3.0) and the informed consent document have been reviewed and approved by the IRBs of the Faculty of Medicine, Universitas Gadjah Mada, Yogyakarta, and Monash University, Melbourne. Any future protocol amendments will be submitted for review and approval by the same IRBs, prior to implementing protocol changes. The trial protocol was registered on ClinicalTrials.gov (NCT03055585) on 14 February 2017.

Confidentiality of participant information will be strictly maintained at all times by the participating investigators, research staff and the sponsoring institution (Universitas Gadjah Mada). This confidentiality is extended to cover testing of biological samples in addition to the clinical, demographic and geospatial information relating to participating subjects. All laboratory specimens, reports, data collection forms and log books, and geolocated records will be identified by a coded ID number only to maintain participant confidentiality. All records that contain names or other personal identifiers, such as informed consent forms, will be stored separately from study records while identified by ID numbers. All local databases will be secured with password-protected access systems. No information concerning the study or the data will be released to any unauthorised third party, without prior written approval of the sponsoring institution. Clinical or personal information will not be released without written permission of the subject, except as necessary for monitoring by an ethical review board or regulatory agencies. Reporting of study results will not be done in any way that permits identification of individual participants, or the location of their homes or other visited locations.

## Discussion

There exists little empirical evidence to support the effectiveness or optimal application of the vector control tools routinely employed to combat arboviruses transmitted by *Ae. aegypti* mosquitoes, including dengue, Zika and chikungunya. The first dengue vaccine has recently been licensed, but has a complex efficacy and safety profile that will constrain its implementation in many endemic settings. A critical need has therefore been highlighted by the scientific community [13] and the WHO [32] for carefully designed trials of existing and novel vector control interventions – with epidemiological endpoints – in order to effectively impact the transmission of dengue and other arboviruses. This study aims to address this need, as the first trial designed to measure the impact of *Wolbachia*-infected mosquitoes on dengue and other arboviral diseases in an endemic setting.

We have employed a novel epidemiological design, the CR-TND [22, 23], which uses outcome-based concurrent sampling of dengue cases and non-dengue controls to measure the efficacy endpoint. This has the potential to be more efficient, cost effective and logistically simpler to achieve than traditional CRT designs with prospective cohorts. An extensive literature on the application of the TND in (non-randomised) studies of influenza vaccine effectiveness demonstrates the validity of effect estimates (odds ratios) from a TND, and their equivalence to direct estimates of relative risk in the source population, provided certain assumptions are met [33–38]; primarily, that the distribution of test-negative illness is not associated with intervention status, and that test-negative controls are allowed to include participants who may be classified as dengue cases at other times during the study period.

Our study uses constrained randomisation, balancing on the historical incidence of undifferentiated febrile illness in clusters, to reduce the risk of a chance imbalance in the distribution of test-negative controls between study arms. However, we cannot exclude the possibility that spatial clustering of a non-arboviral febrile illness will result in a differential distribution of test-negative controls between study arms during the 2-year study period. We will assess this in the trial data by calculating the odds of *Wolbachia* exposure among enrolled test-negative controls, with the expectation that the odds should approximate one if the assumption of no association is upheld.

The spatial and temporal heterogeneity that is characteristic of the dengue epidemiology presents a challenge to the evaluation of dengue control interventions. At a range of spatial scales, including within a city, there exists substantial heterogeneity in the distribution of dengue transmission from year to year [39–46]. This heterogeneity effectively increases the baseline inter-cluster variance, and therefore reduces the power to detect a difference between study arms that can confidently be attributed to *Wolbachia* deployment or conversely increases the number of clusters needed to detect an effect. We were limited by pre-defined boundaries of the study site to having only 24 clusters, given the requirement to maintain a sufficiently minimum cluster size to enable *Wolbachia* establishment and minimise contamination by mosquito and human movement. Nonetheless, our power calculations have taken into account the spatial heterogeneity observed in historical dengue and febrile illness case distributions, and indicate that adequate power is retained. We will re-run these power calculations using real trial data once the trial has begun. An individual-level (per-protocol) analysis will also account for spatial clustering of test-positive or test-negative illness by incorporating a parameter for participants’ cluster.

Inter-annual fluctuations in dengue transmission mean that the study might fall in a period of lower incidence just by chance, and indeed the high dengue incidence seen in Yogyakarta in 2016 makes this a real possibility. However, the efficiency gains of the CR-TND make this less of a concern than it would be for a prospective cohort study, and the target sample size of a minimum of 1000 dengue cases and 4000 test-negative controls is seen as feasible even in the event of two consecutive low-transmission seasons.

The nature of the *Wolbachia* intervention means that blinding was considered infeasible, as it would have doubled the resources and time required to conduct field releases of mosquitoes, for example, with inactive eggs as the placebo. The risks to study validity from a non-blinded deployment (for example, if a belief that *Wolbachia* is protective against dengue cases, residents of treated areas may be less likely to seek care for a febrile illness) were also seen to be minimised by the fact that dengue cases and test-negative controls are drawn from the same patient population, who are clinically indistinguishable at the time of presentation and enrolment at clinics. Thus, the CR-TND is tolerant to the possibility that healthcare seeking behaviour is modified by knowledge of the *Wolbachia* status, as long as this modified behaviour applies equally to test-positive dengue cases and test-negative controls [47].

Human movement is a challenge for cluster randomised trials of community-based interventions applied to geographic areas, because the individuals in whom the efficacy endpoint is measured may spend some proportion of their time outside their cluster of allocation, resulting in exposure misclassification. This has the effect of making the populations in treated versus untreated study arms more similar to each other in their true exposure distribution, and therefore biases the estimation of intervention effect towards the null. We have accounted for this in two ways. Firstly, by powering the study to detect a relatively conservative effect size of 50%, we have allowed for some dilution of effect by human movement. Secondly, by collecting travel history data from the period immediately prior to the onset of febrile illness, we will perform an individual ‘per-protocol’ analysis in which a quantitative exposure status is adjusted both for the time spent in clusters away from home and the local measure of *Wolbachia* prevalence in visited clusters.

*Wolbachia* establishment is likely to be heterogeneous within a cluster due to spatial variation in mosquito populations and dispersal. The classification of individual participants’ *Wolbachia* exposure status will therefore be imperfect even in the per-protocol analysis, because *Wolbachia* prevalence is aggregated from all trapped mosquitoes within one cluster in one 4-week period (from a network of traps at a density of 16 per km^2^). Because this inaccuracy is assumed to be non-differential between clusters, the resulting exposure misclassification should only bias the effect estimate towards the null.

Extensive effort has been invested in local community engagement leading up to this trial, extending from community leaders and key stakeholders to the media and the general public, with an aim of informing the community about the planned *Wolbachia* releases and addressing any concerns. Approval for releases was given by community leaders after extensive community consultation, with individual residents’ consent obtained for hosting an MRC at their property. Community acceptance of the releases has remained high prior to, during and now post-releases, and will continue to be monitored throughout the duration of the trial.

We anticipate that, if the results of this trial do demonstrate a reduction in dengue incidence associated with *Wolbachia* deployment, this should be broadly generalisable to other dengue endemic settings. Any threats to external validity of the study findings will likely relate to the local entomological context that influences the ability of *Wolbachia* mosquitoes to establish and sustain, and thus the specific approach to deployment may need to be tailored to local contexts. The dengue-blocking phenotype of *Wolbachia* is also partly serotype specific, so the local distribution of circulating DENV serotypes in other settings – and also differences in intensity of transmission – may theoretically result in differences in observed disease impact in different settings. A critical next step in translating efficacy trial results into public health implementation of *Wolbachia* will be cost-effectiveness analyses and cost optimisation of different deployment scenarios, so that the findings from this trial can be used to inform decision-making in other settings as efficiently as possible.

### Trial status

At the time of submission, *Wolbachia* releases in intervention clusters have finished and participant recruitment in the clinical study has commenced. Pilot recruitment commenced in November 2017, recruitment into the intention-to-treat dataset commenced in January 2018, and recruitment is expected to be completed by December 2019. The current approved protocol is version 3.0, 13 March 2018.

## Additional file

Additional file 1: SPIRIT Checklist. (PDF 197 kb)

## Abbreviations

CHIKV: chikungunya virus
CR-TND: cluster randomised test-negative design
DENV: dengue virus
IDMC: independent data monitoring committee
IRB: institutional review board
MRC: mosquito release container
NS1: non-structural protein 1
PCR: polymerase chain reaction
RR: risk ratio/rate ratio
RT-qPCR: reverse transcriptase quantitative PCR
TSC: trial steering committee
WEI: *Wolbachia* exposure index
WHO: World Health Organization
